# Case report: TP53 heterogeneous alteration in grade 3 type I gastric NET: a case implicating primary tumor progression

**DOI:** 10.3389/fonc.2025.1708609

**Published:** 2025-12-05

**Authors:** Yan Li, Yuekui Bai, Hongjuan Ti, Chunyu Zhou, Qian Liu, Ying Dong

**Affiliations:** 1 Department of Pathology, Haidian Hospital, Haidian District of Peking University Third Hospital, Beijing, China; 2 Department of General Surgery, Haidian Hospital, Haidian District of Peking University Third Hospital, Beijing, China; 3 Department of Pathology, Peking University First Hospital, Beijing, China

**Keywords:** neuroendocrine tumor grade 3, TP53 mutation, autoimmune metaplastic atrophic gastritis, gastric neuroendocrine tumor (GNET), pathological factors

## Abstract

Grade 3(G3) type I gastric neuroendocrine tumors (NETs) are rare, with only three documented cases in the literature to our knowledge. The case we report involves a type I gastric NET(g-NET) classified as G3, which exhibits heterogeneity in TP53 gene mutations. This suggests that G3 g-NETs may develop from G2 forms. In this tumor located in the gastric body, both G2 and G3 components coexist. The G2 component is found in the superficial mucosa and submucosa, while the G3 component is in the submucosa and muscularis propria. Genetic testing reveals that the TP53 gene is mutated in the G3 component. The coexistence of these alterations within a single primary tumor is extremely rare, indicating that the TP53 gene plays a crucial role in the progression from G2 NET to G3 NET. This case may challenge our previous understanding of g-NETs.

## Introduction

The incidence of gastric neuroendocrine neoplasms (G-NENs) rose from 0.435 to 7.033 per 1, 000, 000 persons over the past 46 years (1). Research indicates that the incidence rate of NENs among the Chinese population is approximately 4.1 per 100, 000 people. The most prevalent sites for these tumors are the pancreas (31.5%), rectum (29.6%), and stomach (27%) (2, 3). In 2022, the World Health Organization (WHO) published standardized classification and grading criteria for epithelial NENs. NETs are subclassified into three grades: G1, G2, and G3, based on mitotic activity and Ki-67 proliferation index (4). High-grade gastroenteropancreatic neuroendocrine neoplasms (GEP-NENs) can be morphologically classified into well-differentiated NETs (G3) and poorly differentiated neuroendocrine carcinomas (NECs). However, there is currently no consensus on the specific morphological criteria for this classification. In well-differentiated neuroendocrine tumors (WD-NETs), grading heterogeneity may be observed either within the tumor itself or among metastatic sites, such as the progression from primary G1 tumors to metastatic G2 tumors. Nonetheless, the progression from Grade 1 or Grade 2 WD-NETs to Grade 3 NETs is rare (5), and such cases have not been reported in type I g-NETs specifically. Thus, further investigation into the histological and molecular characteristics of these tumors is warranted.

## Case presentation

A 63-year-old female was admitted to the hospital due to “intermittent dizziness lasting for one and a half years, and a change in stool color to a darker shade over the past month”. Intermittent dizziness has occurred for the past year and a half. Black stools were first noticed one month ago, and two weeks ago, the patient began to have an increased amount of black stools accompanied by dizziness and palpitations. There were no symptoms such as nausea, vomiting, or hematemesis. The patient has a history of hypertension and hepatitis, with no family medical history. Laboratory tests revealed a hemoglobin level of 72 g/L (normal range: 115–150 g/L). A bone marrow examination at another hospital suggested iron-deficiency anemia, for which the patient received oral iron supplements and intramuscular vitamin B12 injections.

**Figure 1:**
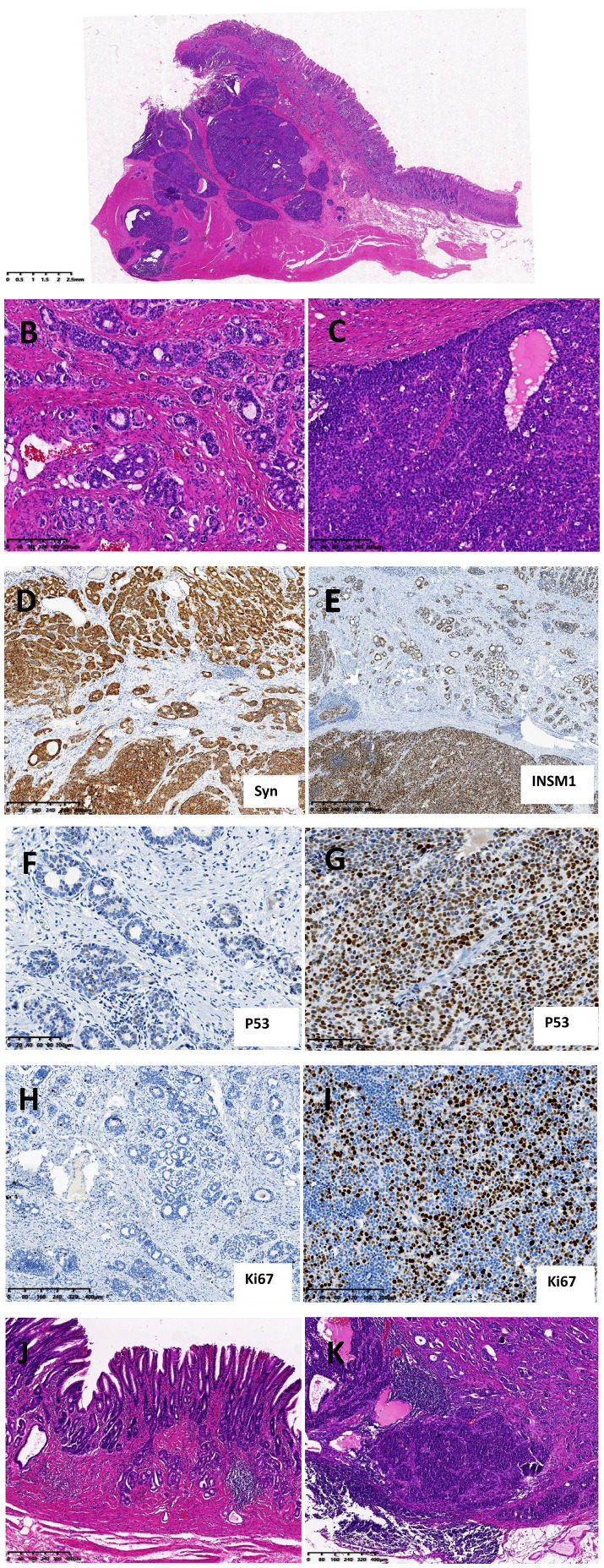
Grade 3 type I gastric NET histological images and partial immunohistochemical **(IHC)** images **(A)** Overview of the tumor. **(B)** G2 NET component, the tumor cells are arranged in glandular, trabecular, and ribbon-like patterns. **(C)** G3 NET component, solid and nested arrangement of tumor cells. **(D)** Syn shows diffuse positivity in both the G2 and G3 components. **(E)** INSM1 also shows diffuse positivity in both the G2 and G3 components. **(F)** The P53 protein exhibits a scattered wild-type expression pattern with varying intensities in the G2 component. **(G)** The P53 protein exhibits a nuclear overexpression pattern in the G3 component. **(H)** Ki-67 positivity in 3-5% of cells in NET G2. **(I)** Ki-67 positivity in 50% of cells in NET G3. **(J)** Pathological Morphology of Autoimmune Metaplastic Atrophic Gastritis (AMAG) in the Gastric Body. **(K)** Tumor lymph node metastases include both G2 and G3.

Gross examination of the surgical specimen, an ulcerative mass measuring 2.2 cm × 2.0 cm × 0.8 cm on the posterior wall of the gastric corpus, near the greater curvature. Microscopic evaluation of the tumor indicated infiltrative growth, affecting the mucosal, submucosal, and muscular layers (**Figure 1A**). The tumor exhibited two distinctly different components: the G2 component, which was in the mucosal and submucosal layers of the gastric wall. The tumor cells were of moderate size, arranged in glandular, trabecular, and ribbon-like patterns, with only a few small foci of solid tumor cell nests observed. The cells were oval or polygonal, displaying pleomorphic nuclei, visible nucleoli, and 4–6 mitotic figures per 2 mm², classifying it as a low-grade malignancy (**Figure 1B**). High-grade component: this part was primarily located in the deeper layers of the gastric wall, specifically the submucosal and muscular layers, and exhibited a nest-like infiltrative growth pattern. The cells in this area were round or polygonal and larger, showing more pronounced cellular atypia, marked nuclear staining, prominent nucleoli, and a higher number of mitotic figures (15–22 per 2 mm²), thus classified as high-grade malignancy (**Figure 1C**). Vascular and perineural invasion were also identified. Both components demonstrated focal intermingling within the submucosal layer. Immunohistochemical staining results were as follows: both components tested positive for Synaptophysin (Syn, **Figure 1D**), Chromogranin A (CgA), and as well as Insulinoma-associated protein 1 (INSM1, **Figure 1E**). Somatostatin Receptor 2 (SSTR2) showed strong positivity in the G2 area and weak positivity in the G3 area. P53 displayed 5% nuclear positivity in the G2 area, while over 70% nuclear positivity was observed in the G3 area (**Figures 1F, G**). Sanger sequencing identified a point mutation (R273H) in the P53 gene in the NET G3 component; however, it is challenging to isolate the NET G2 component alone by microdissection. Interfering peaks make the test data difficult to read. Single-cell sequencing will be used for further clarification when conditions permit. Ki-67 indicated 3-5% positivity in the G2 area and 50% positivity in the G3 area (**Figures 1H, I**). Retinoblastoma protein (Rb) exhibited wild-type expression in the cells of both components. P16 is negative in the tumor cells of both regions but shows partial positive expression in the stromal cells. In the gastric body mucosa, parietal and chief cells were significantly reduced, and gastric fundic glands were diminished to the point of disappearance. A large number of lymphoplasmacytic infiltrates are present in the deep part of the lamina propria of the mucosa. Additionally, gastric pseudopyloric gland metaplasia and intestinal metaplasia were observed. There was also linear and small nodular hyperplasia of neuroendocrine cells. The mucosa of the gastric antrum shows mild chronic gastritis. These findings suggest a diagnosis of autoimmune metaplastic atrophic gastritis (AMAG, **Figure 1J**). Metastasis is identified in 2 lymph nodes, with the metastatic tumor components including G2 NET and G3 NET (**Figure 1K**). Pathological Diagnosis: Gastric neuroendocrine tumor, Grade 3, with vascular and perineural invasion, lymph node metastasis identified, and AMAG in the surrounding gastric corpus. After surgery, the patient underwent chemotherapy with etoposide and cisplatin (EP). One year later, the patient developed liver metastasis and ultimately passed away 18 months after the initial treatment.

## Discussion

In pathology, NENs are subclassified into WD-NETs and poorly differentiated NECs based on the degree of differentiation. The pathological distinction between NETs G3 and NECs has always been challenging. Several studies have indicated consistency in the histological diagnosis between NET G3 and NEC (6, 7). Especially, large-cell NECs frequently overlap with NETs G3 in histomorphology, mitotic figures, and Ki-67 index expression, posing a significant diagnostic dilemma (8). In pathological diagnosis, high-grade NENs containing G1 and/or G2 NET components are more prone to being diagnosed as NET G3. According to the 2022 WHO grading criteria for NENs, the g-NET case we present would be classified as NET G3 rather than NEC. The molecular characteristics of NET G3 and NEC are distinct. GEP-NECs typically show common alterations in the TP53 and RB1 genes (8). Pancreatic NETs frequently exhibit changes in genes such as MEN1, DAXX, ATRX, TSC1, TSC2, CDKN1A, and CDKN1B, along with alterations in CDKN2A and SETD2 in metastatic lesions. Other gastrointestinal NETs (GI-NETs) display different genetic alterations compared to pancreatic NETs, primarily associated with the Notch signaling pathway (9, 10). Prior research has documented mutations in the TP53 gene in high-grade metastatic NETs, while wild-type TP53 has been observed in low-grade primary lesions. This suggests that TP53 mutations may drive tumor metastasis (9, 10). The co-abnormal TP53 and RB1 may reduce the ability of cancer cells to arrest the cell cycle and repair DNA damage, leading to increased reliance on subsequent cell cycle checkpoints. The co-abnormal TP53 and RB1 of Small Cell Lung Cancer may contribute to its sensitivity to DNA-damaging treatment, such as platinum-based chemotherapy (11). Data from multiple previous studies on g-NEC have shown the following: 14 cases (with 64.3% TP53 genetic variation and 21.4% RB1 genetic variation, including copy number variations), 8 cases of gastric NEC (87.5% TP53 genetic alteration and 12.5% RB1 genetic alteration) (12), and 25 cases of gastric NEC, in which the rates of aberrant TP53 and RB1 genetic alterations were 68% and 36%, respectively (13).

Multiple studies recommend using immunohistochemistry to detect P53 and Rb proteins to help differentiate between grade 3 NETs and NECs (14–16). In the case of a G3 gastric NET discussed here, the G2 component is located superficially, while the G3 component is situated deeper within the tissue. This arrangement suggests that the tumor may have originated with better differentiation and subsequently undergone a high-grade transformation during its growth. Immunohistochemical staining supports this idea, showing that the expression of P53 in the G3 component is significantly higher, with 70% of cells testing positive, compared to just 5% in the G2 portion. Although previous studies have shown that P53 protein expression levels can serve as reliable indicators for TP53 gene mutation status and possess predictive value (17, 18). However, in this case report, there is a limitation in inferring that the TP53 gene of the G2 component is wild type solely through immunohistochemistry. The coexistence of wild-type and mutant P53 protein expression in primary NETs is extremely rare, suggesting that P53 plays a critical role in the progression of NETs from low-grade to high-grade. When examining Rb through immunohistochemical staining, there were no significant differences in expression levels noted. According to the literature, rare pancreatic G3 NETs that have progressed from G1/G2 NETs may harbor mutations in MEN1 and ATRX genes, as well as concurrent mutations in TP53 and RB1, hinting at a potential progression from NETs to NEC (16, 19). However, studies focused on g-NETs of this nature are exceedingly rare. In this case, the immunohistochemical analysis revealed alterations in the P53 gene, and the complete absence of P16 protein expression may suggest abnormalities in related genes. The P16 protein indirectly protects the Rb protein from phosphorylation by inhibiting CDK4/6, and together, they form the regulatory pathway for the G1/S phase of the cell cycle. The expression pattern of immunohistochemical P16-/Rb+ has been reported in large cell neuroendocrine carcinoma of the lung and Merkel cell carcinoma of the skin to be associated with treatment and prognosis; however, no literature has been reported on g-NETs (20–22). The expression pattern in this case is P16-/Rb+, and whether it indicates a shorter patient survival time still requires the accumulation of more relevant cases for in-depth discussion. No abnormal expressions were found in ATRX or other genes. The expression characteristics of SSTR2 in GI-NETs vary by primary site. In foregut NETs, SSTR2 immunoreactivity is significantly negatively correlated with the Ki-67 labeling index, while in hindgut NETs, the two show a significant positive correlation (23). Among g-NETs, WD-NETs exhibit strong positive SSTR2 immunolabeling, whereas poorly differentiated high-grade NENs show weakened or absent SSTR2 immunolabeling. The case in this study is fully consistent with this conclusion.

In this case, we must also recognize an important issue: When only WD-NETs are observed in small biopsy specimens, there may still be other poorly differentiated components present, and we must not be “blind to the rest.” Small biopsy specimens make it difficult to assess the grading progression of NETs, and there is likely tumor grade heterogeneity within WD-NETs. Some researchers suggest that grading progression can occur in WD-NETs, and the phenomenon observed in our case supports this conclusion. As we increasingly understand that G2 NET can undergo secondary genetic alterations leading to the development of high-grade tumors, this presents significant challenges for the stratified treatment of NETs. Tumor size, perineural invasion, and TNM stage were independent prognostic factors of gastric high-grade NENs. Previous studies have shown that there is no significant difference in the prognosis between g-NEC and g-NET G3 after radical resection (24).

G -NETs are generally classified into three clinical subtypes. Type 1 g-NETs comprise 80% to 90% of cases and are primarily associated with autoimmune atrophic gastritis. Type 1 g-NETs are predominantly G1 and G2 grade tumors, with G3 grade tumors being rare. Type 2 g-NETs, which account for 5% to 7% of cases, result from excessive gastrin secretion due to gastrinomas. Type 3 g-NETs represent approximately 10% to 15% of cases, and their pathogenesis remains uncertain. In the specific case presented, multifocal infiltration of lymphocytes and plasma cells was observed in the deep layer of the gastric mucosa lamina propria. Additionally, pseudopyloric gland metaplasia (antralization of the fundus and body mucosa) was found in the gastric fundus and body. ECL cells exhibited hyperplasia of varying degrees, confirmed by CgA and Syn immunohistochemistry stain, indicating AMAG. Based on a comprehensive analysis of clinical symptoms, laboratory findings, treatment history, and pathological morphological features, this case is consistent with the diagnosis of Type 1 g-NET. The pathological manifestations of AMAG are categorized into three stages: early stage, florid stage, and terminal stage. Early-stage AMAG can often be indistinguishable from other types of gastritis, such as infectious gastritis, while the terminal stage is frequently confused with atrophic gastritis (25). Furthermore, the endoscopic diagnosis of AMAG has low sensitivity and specificity. Due to a lack of awareness among clinicians and pathologists, its characteristics are frequently overlooked (26). When pathological manifestations of AMAG are identified in gastroscopic biopsy specimens, clinicians should be attentive to laboratory test indicators. These include serum gastrin levels, 24-hour gastric acid output, anti-parietal cell antibody, anti-intrinsic factor antibody, serum vitamin B12, and ferritin levels to avoid a missed diagnosis. In summary, this case presents unique histomorphology, rare expression of molecular markers, and uncommon biological behavior, providing valuable insights for a deeper understanding of g-NETs. The patient in this case received the EP chemotherapy regimen. This regimen has been used in the treatment of various NENs, and studies have suggested that its efficacy in WD-NET G3 is lower than that in poorly differentiated NECs: the objective response rate (ORR) for high-proliferative NECs can reach 44%, while that for NET G3 is only 24%. Additionally, patients with panNET G3 harboring Rb protein loss or KRAS gene mutation have a significantly higher ORR to the EP regimen than those without these characteristics (27).

## Conclusion

Primary type 1 g-NETs can progress from grade G2 to G3, and the TP53 gene plays a crucial role in this process. Given the rarity of such tumors and their susceptibility to misdiagnosis in clinical practice, additional case accumulation and in-depth research are required. Clinical experience in the treatment of gastric NET G3 is limited, so multidisciplinary collaboration is necessary to develop effective personalized treatment regimens for patients.

## Data Availability

The original contributions presented in the study are included in the article/supplementary material. Further inquiries can be directed to the corresponding authors.
